# Characterization of Inferior Rectus Muscle Action in Normal Subjects Using Real-Time Magnetic Resonance Imaging of the Orbit

**DOI:** 10.3390/cmtr19020020

**Published:** 2026-04-05

**Authors:** Alexander R. Engelmann, Kailash Singh, Jiachen Zhuo, Néha Datta, Alfredo A. Sadun, Michael P. Grant, Shannath L. Merbs

**Affiliations:** 1 Department of Ophthalmology and Visual Sciences, University of Maryland, Baltimore, MD 21201, USA; smm8vp@virginia.edu; 2 Department of Oculofacial Surgery, Cole Eye Institute, Cleveland Clinic Foundation, Cleveland, OH 44106, USA; 3 Cleveland Clinic Lerner College of Medicine, Cleveland, OH 44195, USA; singhk11@ccf.org; 4 Department of Diagnostic Radiology, University of Maryland, Baltimore, MD 21201, USA; jzhuo@som.umaryland.edu; 5 Department of Plastic & Reconstructive Surgery, Johns Hopkins School of Medicine, Baltimore, MD 21287, USA; neha@jhmi.edu; 6 Doheny Eye Institute, University of California Los Angeles, Pasadena, CA 90095, USA; alfredo.sadun@gmail.com; 7 R. Adams Cowley Schock Trauma Center, University of Maryland, Baltimore, MD 21201, USA; wjz3gc@uvahealth.org; 8 Department of Ophthalmology, University of Virginia, Charlottesville, VA 22903, USA

**Keywords:** dynamic orbital anatomy, real-time MRI, inferior rectus morphology, eye movement

## Abstract

Orbital floor fractures may cause long-term functional and esthetic impairments. Diplopia due to impaired function of the inferior rectus muscle is frequently an indication for surgical repair, but some cases, such as those where the diagnosis has been delayed or a previous attempt at repair has been made, may not always be amenable to surgical correction. It is advantageous for the surgeon to know whether the proper function of the inferior rectus muscle can be restored for the purposes of surgical planning and prognostication. The authors hypothesized that real-time MRI could be used to characterize the appearance of the inferior rectus muscle in a way that would facilitate future analysis of inferior rectus function in patients with diplopia due to orbital floor fractures. Real-time MRI was performed on 10 volunteer participants with normal ophthalmic function and orbital anatomy to assess inferior rectus appearance during vertical duction testing. ImageJ software was used to measure and record characteristics of the inferior rectus muscle, viewed in a quasi-sagittal plane. The ratios evaluated included inferior rectus muscle length in upgaze versus downgaze (UDR, mean 1.58) as well as inferior rectus muscle length versus distance from inferior rectus origin to inferior rectus inflection point in upgaze (LIR, mean 1.30) and downgaze (mean 1.20). These values were found to be conserved between orbits and individuals. This data offers quantitative insight regarding inferior rectus muscle appearance across the full arc of vertical gaze in healthy individuals. We plan to use this normative baseline dataset as a comparison for future phases of this project, using real-time MRI to evaluate traumatized orbits with diplopia and derangement of the inferior rectus muscle.

## 1. Introduction

Orbital floor fractures can result in long-term functional and esthetic impairments; therefore, identifying patients who are likely to benefit from operative intervention is critical to prevent significant morbidity. Derangements of the inferior rectus muscle have been well-reported in patients with diplopia due to upgaze difficulties following orbital floor fractures [1]. Common indications for repair of isolated orbital fractures within 2 weeks of injury are large fractures resulting in acute enophthalmos and orbital tissue entrapment causing diplopia. However, the decision to perform delayed or revisional surgery in patients with diplopia is more nuanced. For example, is the diplopia in primary gaze or only extreme gaze? Is the etiology of the diplopia a tethered muscle in the fracture or an improperly placed implant that may benefit from surgery or is the motility limitation inherent muscular fibrosis resulting from the injury or prior surgery that would be unlikely to improve with additional surgery? We hypothesized that observing the orbital tissues in real time during duction testing would improve our understanding of post-traumatic ocular motility disturbances and help to discern between these two situations. While standard computed tomography (CT) imaging, routinely obtained in the case of orbital fractures, is ideal for assessing bony injury and provides adequate information in the majority of circumstances, Magnetic Resonance Imaging (MRI) is a superior modality for characterizing soft tissues [2,3]. Our phase-one project aimed to quantitatively characterize real-time changes in extraocular muscles and orbital tissues in healthy subjects to establish a normative baseline dataset, to compare with future subjects with diplopia due to orbital floor fracture with delayed presentation or persistent diplopia despite prior fracture repair [4].

Given that orbital floor fractures are the most commonly encountered clinically [5], and inferior rectus (IR) muscle dysfunction is the most likely culprit when a patient has vertical diplopia after an injury, we identified two ratios of interest to define among healthy individuals: the ratio of the length of the IR in most extreme upgaze versus most extreme downgaze (UDR) and ratio of the length of the IR to the distance from the IR origin to the IR inflection point (LIR), which can be quantified in any defined position of gaze [6]. The extraocular muscles take a unique course between their origin at the annulus of Zinn and insertion on the globe, being inflected by connective tissue sheaths which form pulleys that protect the muscle from orbital fat and influence the muscle’s functional insertion and arc of contact [3]. The location of the inferior rectus inflection point has been previously characterized with MRI and anatomically [6,7]. It is seen at a mean distance of 6 mm posteriorly and 12.9 mm inferiorly relative to the center of the globe. Given its relation to the globe and its influence on ocular motility, we expect its dynamic location to be a viable proxy for the functional status of the inferior rectus and adjacent orbital connective tissue. Specifically, we predict that the location of this inflection point will be disrupted in orbits with floor fractures that are causing diplopia. Using the ratio of the inflection point to the length of the muscle allows for comparison between individuals who have larger or smaller orbits. We intend to use these quantitative parameters to define the qualitative differences that we might encounter with a traumatically deranged inferior rectus muscle and orbital soft tissues, such as rounding of the inferior rectus muscle [8,9]. The data obtained from this study should allow us to correlate imaging with clinical findings, guiding operative intervention specifically for delayed fracture repairs or revisions in diplopic patients for the planned phase two of the study.

## 2. Materials and Methods

The University of Maryland Institutional Review Board (IRB) provided approval and oversight for this study, which was conducted in accordance with the tenants of the Declaration of Helsinki. Written proof of informed consent was obtained. Of the 10 total participants, 7 were recruited for this non-blinded experimental study from a prior study which assessed binocular function, and the remaining 3 participants were recruited using university-approved distributed advertisements. Subjects qualifying for inclusion were 18 years of age or older and capable of lying flat in an MRI scanner for up to 1 h. Exclusion criteria were a prior history of ophthalmic pathology or surgery as well as prior ocular or orbital trauma. All participants were required to complete a complete ocular function assessment and a detailed pre-MRI safety screening checklist with questions regarding any prior diagnostic imaging or examination, surgery, implants, medications, or possible foreign bodies.

**Figure 1:**
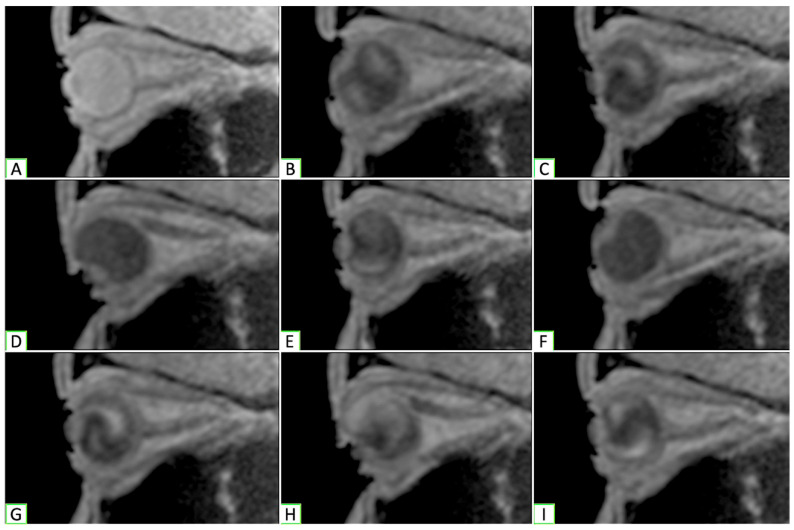
Dynamic orbital MRI (Vedio S1) cuts from each position of gaze analyzed. (**A**,**C**,**E**,**G**,**I)** Primary; (**B**,**F**) upgaze; (**D**,**H**) downgaze.

Real-Time Magnetic Resonance Imaging (rtMRI) was carried out with a 3-Tesla Siemens MAGNETOM Prisma MRI scanner (Malvern, PA, USA) augmented with 8-channel phased-array coils. Using scout images, an oblique-sagittal plane centered on the apex of the cornea and the apex of the orbit was selected to obtain rtMRI data from each orbit while the participant moved their eyes between primary, up, and downgaze. A gradient echo FLASH (fast-low angle shot) sequence was used to obtain iterative images in a single plane. A total of 90 frames were captured over a 77 s interval, which equated to two periods of oscillation per eye, providing a total of eight movements to be analyzed per eye (Figure 1).

The fellow eye was occluded with an opaque plastic shield to eliminate any convergence stimulus. The right eye was imaged first, followed by the left eye. Prior to entering the scanner, participants were briefed on how and where they should direct their eyes during the dynamic portion of testing. Participants were told to gradually shift their gaze as indicated by the screen, spending no more than 10 s moving to each position of gaze. During the test, participants received verbal instructions as well as written instructions which were broadcast on a monitor positioned at the head opening of the scanner, visible to participants by use of a mounted mirror. Since participants would not be able to read the screen while following instructions, a simple color-coded system was used.

**Figure 2:**
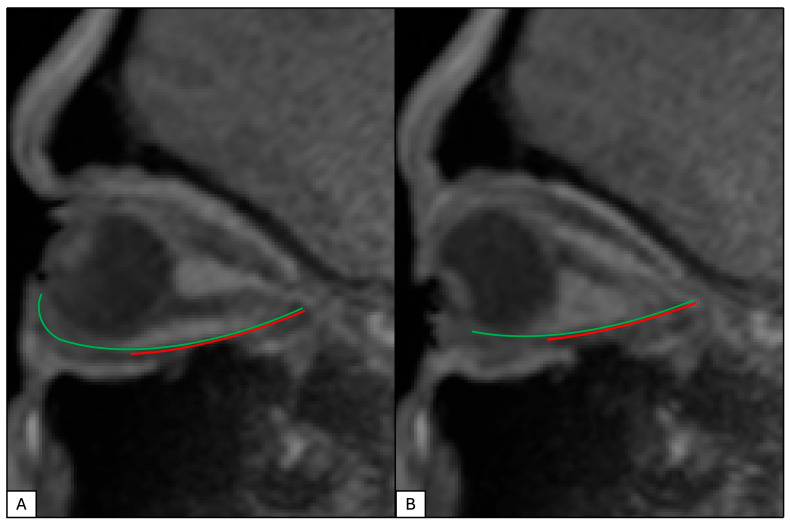
Illustration of inferior rectus length measurement. Inferior rectus muscle origin to insertion (green) and inflection point (red) in (**A**) upgaze and (**B**) downgaze.

Image analysis was performed on a Macbook Pro (Cupertino, CA, USA) 14-inch Hi-DPI 3024 × 1964 pixel display using the ImageJ graphical user interface version 1.53t, which is publicly available from the National Institutes of Health (Bethesda, MD, USA). Only images free of artifacts and degradation were used for quantitative analysis. Measurements were considered certain to one tenth of a millimeter. The inferior rectus (IR) muscle of each subject was measured from its origin at the orbital apex to insertion in the most extreme up and downgaze. The origin of the IR was approximated by marking the point at which the superior rectus, inferior rectus, and optic nerve appear to converge, the annulus of Zinn (Figure 2). The IR tendon insertion site on the globe was estimated by measuring approximately 6.5 mm inferior to the location of the inferior corneal limbus, the approximate point at which the IR tendon inserts according to the spiral of Tillaux. The insertion points of the rectus muscles certainly vary between individuals, but no study has convincingly asserted any specific relationship between an easily ascertained value (such as axial length) and insertion distance. The most minor susceptibility artifact can obscure the insertion point, making its measurement only certain to the 1.0 mm, rather than 0.1 mm as is required for this study. Therefore, the location defined by the clinically applicable spiral of Tillaux was considered the most exact and repeatable method to determine the insertion point for the inferior rectus muscle. The distance from the origin to the inflection point of the IR (a point where the connective tissue pulley system inflects the muscle path away from its anatomical origin at the annulus of Zinn) was also measured in the most extreme up- and downgazes (Figure 2) [6]. Additionally, the axial length of the globe was measured in the neutral gaze from the anterior cornea to the inner edge of the retina.

## 3. Statistical Analyses

For each subject and eye, we computed: (i) the ratio of IR length in the most extreme upgaze to downgaze (UDR) and (ii) the ratio of IR length to the origin inflection point distance (LIR) separately in extreme up and downgazes (Figure 2). All ratios were log-transformed prior to analysis to stabilize variance and allow interpretation of effects as multiplicative changes on the ratio scale.

For each ratio type, we fit a linear mixed effects model (LMM) with subject as a random intercept (to account for the two eyes measured within each person) and eye (right/left) as a fixed effect. Models were estimated by restricted maximum likelihood (REML), and Wald standard errors were used for inference. Results were back-transformed and reported as geometric means and fold changes on the ratio scale.

Systematic bias was tested via the fixed eye effect. We reported the right–left fold change and its *p*-value. Eye-to-eye scatter was quantified by the residual standard deviation (SD) on the log scale. For clinical interpretability, we reported the corresponding 95% within-subject fold change. In comparing the ratios of IR length to distance from the origin to the inflection point in downgaze, there was near-zero within-subject variability, and the LMM exhibited numerical singularity; therefore, a paired t-test on right–left log differences was used to assess any systematic left-to-right bias. To visualize agreement, we produced Bland–Altman plots on the log scale using the right–left log difference.

To determine consistency between individuals, we used the random intercept to summarize how much subjects differ in their typical ratio after averaging across eyes. We reported the geometric SD and the 95% across-subject fold change and visualized agreement using caterpillar plots with a 10% equivalence band around the mean depicting each subject’s typical ratio and 95% confidence interval. We also calculated the interclass correlation (ICC) to evaluate the between-subject variability relative to the between-eye variability.

To determine whether sex or orbital morphology account for any between-subject variance in IR UDR and LIR, we used a linear mixed-effects model with a random intercept for subject and fixed effects for eye (L/R) plus the covariate of interest—either axial length or gender. Wald tests and 95% CIs REML fits were used for inference.

To assess whether our sample size was sufficient to establish normative values for UDR, and upgaze and downgaze LIR, we performed post hoc power calculations in G*Power (v3.1) with a variance-against-constant analysis using the χ^2^ distribution (one-sided, α = 0.05). Effect sizes were specified on the log-ratio scale to match the analysis, with 20% as our target for clinically meaningful variability.

## 4. Results

In the upgaze, the IR had a mean length of 56.9 mm (range: 51.0 mm–61.1 mm) in the right eye and 56.8 mm (range: 50.7 mm–62.1 mm) in the left, with the mean distance from origin to inflection point measured at 43.5 mm and 44.0 mm (Table 1). The mean length of the IR in the most extreme downgaze was 36.1 mm (range: 34.2 mm–39.0 mm) and 36.1 mm (range: 34.2 mm–38.8 mm) in the right and left eyes, respectively, while the mean distance from the origin to the inflection point was 30.4 mm and 30.2 mm in the right and left eyes (Table 1).

**Figure 3:**
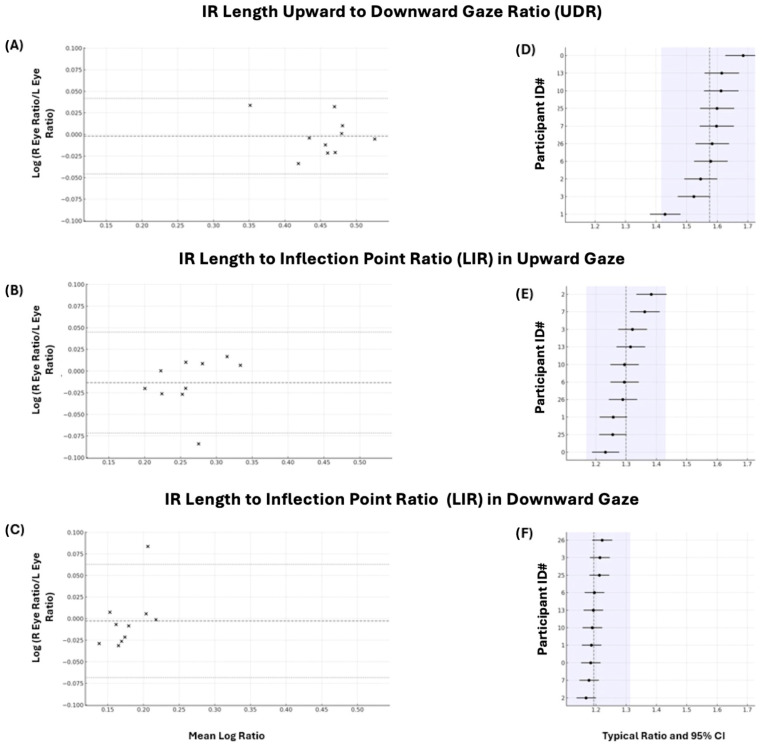
Between-eye agreement and between-subject consistency for IR length ratios. Panels (**A**–**C**) are Bland–Altman plots of the log right-/left-eye difference against the mean log ratio; panels (**D**–**F**) rank subjects by their mixed-model predictions (dots, 95% CIs) with the population geometric mean (dashed line) and a ±10% band (shaded). (**A**,**D**): ratio of upgaze to downgaze IR length (UDR); (**B**,**E**): ratio of upgaze IR length to origin inflection point distance (LIR) (**C**,**F**): ratio of downgaze IR length to origin inflection point distance (LIR).

The mean ratio of the upgaze IR length to the downgaze IR length (UDR) was 1.58 (95% CI 1.53–1.63) in the right eye and 1.58 (95% CI 1.52–1.63) in the left eye. The output of the linear mixed-effects model showed a SD on the log scale of 0.0157 between the eyes, indicating very small within-subject noise. Consistent with this, the estimated right-to-left fold change was 1.002 (Wald *p* = 0.78), demonstrating no systematic L–R bias. Using the residual SD, we calculated the Bland–Altman 95% limits of agreement (LOA) on the ratio scale to be approximately 0.96 to 1.05, implying that for ~95% of subjects, the right-eye ratio is within about ±4% of the left-eye ratio (Figure 3A).

The mean ratio of the upgaze IR length to the inflection point length (LIR) values were 1.29 (95% CI 1.25–1.34) for the right eye and 1.31 (95% CI 1.27–1.35) for the left eye, while the mean downgaze LIR values were 1.19 (95% CI 1.16–1.22) for the right eye and 1.20 (95% CI 1.18–1.21) for the left eye (Table 1). There was a high degree of between-eye agreement in the IR-length-to-inflection-point ratios (LIRs). In upgaze, the right-to-left fold change was 1.013 (Wald *p* = 0.155) with a residual SD (log) of 0.021, which corresponds to a Bland–Altman LOA for the right–left ratio of approximately 0.94–1.06 and a within-subject 95% fold range of about ±4% (Figure 3B). In downgaze, the right-to-left fold change was 0.997 (*p* = 0.80) with a residual SD (log) of 0.016, giving an LOA of roughly 0.956–1.047 and a within-subject 95% fold range of about ±3% (Figure 3C). Together, these results indicate no systematic L–R bias and tight eye-to-eye agreement for this metric in both gazes.

Our measurements demonstrate that in healthy subjects exerting maximum effort, the UDR (upgaze IR length to downgaze IR length ratio) is consistent between individuals. The linear mixed-effects model estimated a between-subject SD (log) of 0.0449, which translates to a 95% across-subject fold range within ±9% of the population mean, coupled with a high intraclass correlation of 0.87 and the absence of any outlying subjects in the caterpillar plot (Figure 3D), demonstrating that the UDR is highly consistent between individuals in this sample.

Our measurements also show that the LIR of the IR length to origin inflection point is consistent among healthy individuals in both gaze directions. In upgaze, the linear mixed-effects model produced a between-subject SD (log) of 0.0385, which corresponds to a 95% fold range within ± 8% of the population mean. The related intraclass correlation was 0.77, and no outliers appeared on the caterpillar plot (Figure 3E). In downgaze, the model estimated an even smaller between-subject SD (log) of 0.0253, resulting in a 95% fold range within ± 5%; the ICC was 0.70, and the caterpillar plot (Figure 3F), likewise, shows no outliers. Overall, these results suggest that the LIR stays tightly clustered between individuals in both gaze directions.

Across subjects, axial length was not significantly associated with any dynamic IR characteristic. For UDR the fold change was 0.998 per mm (95% CI 0.978–1.018, *p* = 0.84). For the upgaze LIR the effect was 1.044 per mm (95% CI 0.967–1.128, *p* = 0.30). For the downgaze LIR, the estimate was 0.993 per mm (95% CI 0.984–1.001, *p* = 0.12), indicating a non-significant tendency toward lower ratios with longer eyes.

Gender was also not associated with any of our dynamic IR characteristics. For all three outcomes—UDR, upgaze LIR, and downgaze LIR—the estimated gender effect was non-significant (UDR: 95% CI 0.978–1.096, *p* = 0.27; upgaze LIR: 95% CI 0.913–1.455, *p* = 0.27; downgaze LIR: 95% CI 0.972–1.032, *p* = 0.91).

Using a post hoc χ^2^ variance-against-constant analysis, we found very high power to show that variability is below a 20% precision target on the ratio scale. The achieved power was 0.89 for UDR, 0.98 for the upward gaze LIR, and 0.99 for the downward gaze LIR. These values reflect that the observed between-subject SDs on the log scale are far smaller than the SD implied by a 20% target, indicating that our study is well powered to demonstrate that dispersion of these metrics is comfortably within the 20% precision threshold.

## 5. Discussion

This data offers a normative characterization of IR measurements across the full arc of vertical gaze in healthy individuals, which we anticipate will be a clinically useful comparison for future phases of this project and other projects evaluating dynamic action of the orbital tissues. We intend to provide a quantifiable explanation for qualitative observations, such as rounding of the inferior rectus muscle or orbital tissue tethering [8,9]. Interestingly, the most tightly clustered value we observed was the LIR (ratio of IR length to inflection point) in downgaze (Figure 3F), while the value with the most variability between individuals was the UDR (ratio of upgaze IR length to downgaze IR length). The authors predict that LIR in downgaze will be a metric that is sensitive to derangements in IR anatomy, as seen when there is herniation of the extraocular muscle and orbital contents into the maxillary sinus following a fracture or a hematoma involving the inferior rectus or nearby tissues [5]. We predict that the difference in LIR value between maximum upgaze and maximum downgaze will differ significantly depending on whether the inferior rectus is merely displaced, acutely tethered, or fibrosed, allowing for better prognostication when planning delayed or revision floor-fracture repairs in patients with diplopia. We predict the displaced or acutely tethered rectus to have a preserved range of LIR between maximum upgaze and downgaze, while the fibrosed rectus will have a significantly reduced range of LIR.

We observed that neither gender nor axial length account for meaningful variation in UDR or LIR in either vertical gaze direction, suggesting that these ratios are consistent across individuals of different orbital morphology. Therefore, adjustment for gender and axial length may be unnecessary when using these ratios diagnostically and deviations beyond the normative reference bounds can be interpreted as biologically relevant rather than demographically or anatomically confounding. As we refine our ability to analyze these scans with finer detail and consistency, we expect to find significant relationships between orbital tissue anatomy and factors such as ethnic background or body type [10], which would impact the diagnostic use of such tools and highlight the importance of plurality within our normative datasets.

It should be clear that the application of this imaging modality is limited to participants who are capable of positioning and lying still in a scanner without sedation, as they will be required to look up and down during the assessment. One feature of this data that should be obvious is its’ sensitivity to participant effort, as smaller excursions in either up or downgaze will produce UDR values closer to 1.0. Despite the relative consistency seen in this dataset, the effort-dependent nature of UDR disqualifies such a metric from being useful as a discrete normative value. It seems most applicable as an index for comparing other values, meaning that other measurements, such as LIR, may be compared to normal ranges which are categorized according to associated UDR values.

One finding that is qualitatively noticeable when reviewing this data is the fact that participants held their most extreme positions of gaze for variable lengths of time. This is in part due to the instructions they were given prior to testing, but also due to the discomfort and visual strain that participants reported experiencing while attempting to hold their most extreme gaze for several seconds. This fact highlights the value of using a real-time imaging sequence, which records a frame every 0.86 s. This meant that participants needed only to hold their eyes in what they consider to be the most extreme up or downgaze for less than 1 s, and as long as they did so at some point during the 77 s imaging run, their orbital anatomy in that position was recorded. Future phases of our project will aim to refine our imaging technique and enhance clinical translatability by employing advanced real-time MRI techniques with an even shorter temporal resolution in addition to employing standardized fixation and gaze targets as well as eye tracking and end-gaze confirmation. One persisting challenge of this imaging modality is the effect of gravity and the supine position that participants assume in order to be scanned, as daily life is experienced with the head upright.

At this stage, it is our expectation that the anatomical relationships that we describe will prove useful for distinguishing fibrosed inferior rectus muscles from viable inferior rectus muscles in a clinically meaningful way, specifically in patients with persistent diplopia following orbital floor fracture either with delayed presentation or prior orbital fracture repair. A notable limitation is the human error introduced while making the measurements in the image-viewing graphical user interface (ImageJ). Future work should include further standardization of the measurement process in order to minimize the influence of human variability and may involve the introduction of machine learning. Fortunately, the data gleaned in this study is far from exhausted and may be analyzed further for additional characterization of normal orbital anatomy. There are many alternative methods for analyzing this data, so a combination of creativity and an aim for repeatability will be key guiding forces. As we move from the qualitative usefulness of a dynamic orbital scan to quantitative analyses with prognostic implications, it is important that anatomical and exploratory studies continue to be performed using the highest quality images to ensure maximum fidelity and reliability as baseline data.

The full potential of dynamic MRI is currently far from realized, as its quantitative clinical use remains limited. This is in part due to the time and resources required for such testing, but as our understanding of dynamic orbital anatomy and techniques such as dynamic orbital computed tomography improve we will likely find broader acceptance and integration into specific clinical practices [11]. We aim to demonstrate such usefulness in the second phase of our project which will compare the characteristics of the extraocular muscles and orbital soft tissues in the setting of isolated orbital floor fractures to the characteristics described in this study, specifically predicting the likelihood of a successful outcome for delayed or revision orbital fracture repairs in patients with diplopia due to inferior rectus derangement. Once such usefulness is demonstrated, we intend to investigate additional applications with disease processes involving tumors or inflammation of the extraocular muscles and orbital soft tissues.

## Figures and Tables

**Table 1:** Participant characteristics and raw orbital measurements.

			IR Length (mm)		Distance from IR Origin to Inflection Point (mm)		
Participant ID #	Gender	Eye (Right/ Left)	Upward Gaze	Downward Gaze	Upward Gaze	Downward Gaze	Axial Length (mm)
0	M	Right	57.7	34.2	47.7	29.2	24.1
		Left	58.0	34.2	47.0	29.0	24.0
1	F	Right	51.0	35.3	41.3	30.4	23.2
		Left	50.7	36.3	40.0	30.3	23.3
2	M	Right	57.0	37.0	40.7	32.7	27.3
		Left	58.0	37.5	41.7	32.2	27.7
3	M	Right	56.5	37.8	42.5	29.5	25.4
		Left	57.2	37.0	43.4	31.4	25.3
6	F	Right	55.7	35.5	43.5	29.8	25.4
		Left	55.9	35.3	42.8	29.3	25.6
7	F	Right	57.2	35.2	41.4	30.1	26.5
		Left	56.8	36.1	41.8	31.1	26.5
10	M	Right	55.9	34.6	43.0	29.6	23.4
		Left	55.2	34.2	42.9	28.5	23.4
13	M	Right	57.2	35.2	45.3	29.9	23.4
		Left	56.3	35.0	41.0	29.1	23.3
25	M	Right	58.6	37.0	46.9	30.1	22.9
		Left	58.7	36.3	47.0	29.7	22.8
26	F	Right	61.1	39.0	48.1	31.4	22.9
		Left	62.1	38.8	47.6	31.2	22.8
Mean	60% M	Right	56.79	36.08	44.04	30.27	24.44
		Left	56.89	36.06	43.52	30.18	24.47

## Data Availability

The datasets presented in this article are not readily available because the data are **part** of an ongoing study. Requests to access the datasets should be directed to the corresponding author (ARE).

## Supplementary Materials

The following supporting information can be downloaded at: https://www.mdpi.com/article/10.3390/cmtr19020020/s1, Video S1: Dynamic Orbital MRI.
